# Differences in salicylic acid glucose conjugations by UGT74F1 and UGT74F2 from *Arabidopsis thaliana*

**DOI:** 10.1038/srep46629

**Published:** 2017-04-20

**Authors:** Alayna M. George Thompson, Cristina V. Iancu, Kenneth E. Neet, John V. Dean, Jun-yong Choe

**Affiliations:** 1 Department of Biochemistry and Molecular Biology, Rosalind Franklin University of Medicine and Science, The Chicago Medical School, 3333 Green Bay Road, North Chicago, IL 60064, USA; 2 Department of Biological Sciences, DePaul University, 2325 N. Clifton Ave, Chicago, IL 60614, USA

## Abstract

Salicylic acid (SA) is a signaling molecule utilized by plants in response to various stresses. Through conjugation with small organic molecules such as glucose, an inactive form of SA is generated which can be transported into and stored in plant vacuoles. In the model organism *Arabidopsis thaliana,* SA glucose conjugates are formed by two homologous enzymes (UGT74F1 and UGT74F2) that transfer glucose from UDP-glucose to SA. Despite being 77% identical and with conserved active site residues, these enzymes catalyze the formation of different products: UGT74F1 forms salicylic acid glucoside (SAG), while UGT74F2 forms primarily salicylic acid glucose ester (SGE). The position of the glucose on the aglycone determines how SA is stored, further metabolized, and contributes to a defense response. We determined the crystal structures of the UGT74F2 wild-type and T15S mutant enzymes, in different substrate/product complexes. On the basis of the crystal structures and the effect on enzyme activity of mutations in the SA binding site, we propose the catalytic mechanism of SGE and SAG formation and that SA binds to the active site in two conformations, with each enzyme selecting a certain binding mode of SA. Additionally, we show that two threonines are key determinants of product specificity.

Salicylic acid (SA) is a plant hormone involved in regulating plant stress responses including local and systemic pathogen responses; UV-C stress, osmotic stress, high light stress, heat, heavy metals, drought, and chilling^1^,^2^,^3^,^4^,^5^,^6^. Depending on the plant species, it has also been shown that SA has additional roles in controlling plant growth and development such as seed germination, growth, flowering, senescence, photosynthesis and respiration^6^. Because of its importance in the physiology of plants, there is a great deal of interest in how plants control the activity of SA. Levels of SA increase during certain physiological responses^7^, however, the role of metabolism and cellular compartmentalization in the control of SA activity is not as well studied.

SA is a phenolic compound (2-hydroxybenzoic acid, Fig. 1a). Because of its reactivity and lipophilicity, small hydrophilic molecules are often added to SA to aid in transport and storage. One example of this conjugation is the formation of SA: glucose conjugates, of which two forms are possible: either an SA-glucoside (SAG) – where glucose is conjugated to the hydroxyl group, or an SA glucose ester (SGE) – with glucose attached to the carboxylate group^8^,^9^,^10^,^11^,^12^,^13^,^14^ (SAG and SGE structures, Fig. 1a). In *Arabidopsis thaliana*, SA glucosylation *in vivo* is performed by two UDP-glucosyltransferase (UGT) enzymes: UGT74F1 and UGT74F2^15^,^16^.

Deletion of these two enzymes at the gene level results in different phenotypes in response to bacterial infection. Lower levels of SA and lower levels of resistance to bacterial infection from *Pseudomonas syringae* are found in *ugt74f1* mutants. In contrast, *ugt74f2* mutants have higher levels of SA and higher levels of resistance to *P. syringae*. Likewise, overexpression of UGT74F2 (also annotated as AtSGT1) results in lower levels of SA and increased susceptibility to *P. syringae*^17^,^18^. As a result of their ability to glucosylate SA, UGT74F1 and UGT74F2 are involved in controlling the levels of “free” SA and, thus, the response of plants to pathogens. Clearly there is interplay between SA, SAG and SGE during a plant’s defense response and which conjugate is formed will have a dramatic effect on the strength of the response.

UGT74F1 will only form SAG from SA and UDP-glucose, while UGT74F2 will form both SGE and SAG with the specific activity for SGE formation being about 10-fold greater than that of SAG formation *in vitro*^15^. UGT74F1 and UGT74F2 will form other products *in vitro*, with both enzymes forming anthranilate-glucose ester^19^, benzoic acid glucose ester and 4-hydroxybenzoic acid glucose ester^15^ and multiple quercetin glucosides^20^. *In vivo* evidence indicates that UGT74F2 produces anthranilate glucose ester^19^.

UGT74F1 and UGT74F2 belong to a family of enzymes with members widely distributed throughout plants: *A. thaliana* contains ~100 UGTs, while *Malus domestica*, the domesticated apple tree, contains ~300^21^. UGTs are known to be important in the metabolism of a number of plant hormones, phenylpropanoids, flavonoids, betalains, coumarins, terpenoids and steroids, glucosinolates, and others^22^,^23^; UGTs directly glucosylate various small molecules, including phenolic compounds, indoles, furans, isoflavones and flavonoids^24^,^25^,^26^. These enzymes are central to plant metabolism with diverse roles that include, among others, pollination, flower and fruit pigmentation, seed dispersal, and plant-pathogen interactions^22^.

From previous structural, biochemical, and sequence analysis, the general organization and activity of these enzymes are known^27^. UGTs are two-domain GT-B proteins with ligand binding sites located in the cleft between domains. UDP-glucose binds to a conserved site, while the glucose acceptor binds to a variable site composed primarily of residues from the N-terminal domain^27^. In addition to the conserved nucleotide site, there are two conserved residues in the N-terminal domain, a histidine and an aspartate that are involved in catalysis. UGTs are inverting enzymes, with their products having opposite stereochemistry at the anomeric carbon to the starting UDP-glucose substrate^26^,^28^,^29^.

As of this writing, there are crystal structures of six plant UGTs deposited in the Protein Data Bank (15 total structures counting the different ligand complexes^26^,^28^,^30^,^31^,^32^,^33^,^34^, see Supplementary Table S1). The previously crystallized proteins display low sequence identity with UGT74F1 and UGT74F2 (26–30%), and do not recognize SA as a substrate, instead producing glucosides of flavonoids, isoflavanones or chlorinated phenols (Supplementary Table S1).

From the UGT structures and biochemical data, it is known that glucoside products are formed via an S_N_2 reaction with a conserved histidine acting as a general base to deprotonate a hydroxyl, which then attacks the anomeric carbon of the nucleotide sugar donor^26^,^33^,^34^. UGTs that form glucose esters (including UGT74F2) are less well studied than those that form glucosides, and their catalytic mechanism is unknown.

In this work, we expressed and purified recombinant UGT74F1, UGT74F2 and various mutants to determine the structural elements responsible for their observed activity differences. UGT74F1 and UGT74F2 are similar (77% protein sequence identity) but produce different products from the same substrates. We solved the crystal structures of UGT74F2 in complex with UDP, UDP and SA, or UDP and 2-bromobenzoic acid (2-BA, an SA analogue), and UGT74F2_T15S_ in complex with UDP and SA or UDP and 2-BA. The crystal structures, along with the activity assays of UGT74F2 and UGT74F1 mutants, suggest that SA can bind in two different conformations, with each enzyme preferring a particular SA binding mode. Additionally, two threonine residues: Thr 15 in UGT74F2 and Thr 365 in UGT74F1, are crucial in selecting the enzyme-specific SA binding conformation and, thus, in product specificity. Finally, the conserved catalytic His 18 is essential for the activity of both UGT74F1 and UGT74F2. The enzyme activity pH dependence and bonding interactions in the active site suggest that the catalytic His 18 is protonated in UGT74F2 to form SGE, while SAG formation in UGT74F1 requires the deprotonated His 18.

## Results

### Recombinant UGT74F1 and UGT74F2 display product specificity

**Figure 1:**
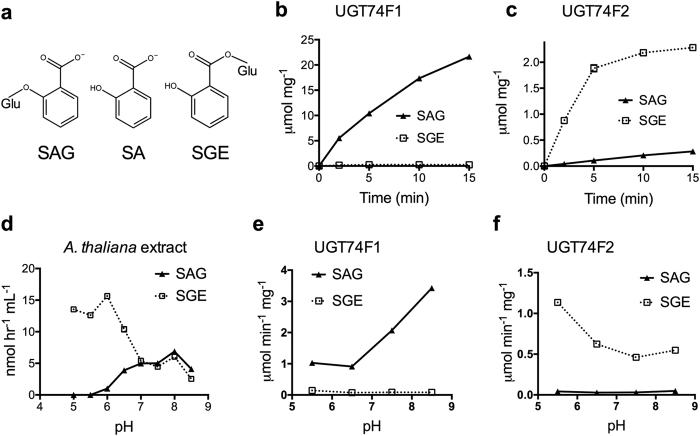
Enzymatic formation of SAG and SGE. (**a**) Structures of salicylic acid (SA), salicylic acid glucose ester (SGE), and salicylic acid glucoside (SAG); Glu represents the glucosyl moiety. Time-course product formation by the recombinant purified UGT74F1 (**b**) and UGT74F2 (**c**). The assays contained 1 μg protein, 5 mM UDP-glucose, 1 mM [7-^14^C]SA (1.5 μCi μmol^−1^), 50 mM Tris-HCl (pH 7.0), and 14 mM 2-mercaptoethanol in a total volume of 200 μL. (**d**) Formation of SAG and SGE in *A. thaliana* cell lysate over pH changes. The assay media included 50 μL cell lysate, 75 mM buffer, 10 mM UDP-glucose, and 0.14 mM [7-^14^C]SA (55 μCi μmol^−1^) in a total volume of 65 μL. pH-dependence of product formation by recombinant purified UGT74F1 (**e**) and UGT74F2 (**f**). Assays were performed as in (**b**) and (**c**) but in buffers of different pH (**e** and **f**). Except for (**b**) and (**c**) all assays are at 3 min time point. (**b**–**f**) Each data point represents the average of 3 measurements.

UGT74F1 and UGT74F2 were expressed recombinantly in *Escherichia coli* and purified to homogeneity for study. *In vitro* activity assay for formation of SA glucose conjugates was adapted from previous studies^16^. Recombinant proteins were mixed with UDP-glucose and ^14^C-SA, conjugates were separated on an HPLC, and quantified by scintillation counting (Fig. 1b and c). UGT74F1 catalyzes production of SAG over the entire 15-minute assay, but negligible SGE is formed (Fig. 1b). The formation of SGE by UGT74F2 increases for five minutes, and then plateaus for the remaining assay time, while SAG formation linearly increases for the entire assay time (Fig. 1c). Interestingly, at one minute UGT74F1 displays a 10-fold higher specific activity for SAG formation than UGT74F2 does for SGE formation (Fig. 1b and c) at pH 7.0.

### SAG and SGE formation is pH dependent

Given previous knowledge that a histidine residue is crucial to formation of glucosides^26^,^33^,^34^, we theorized that protonation state of the catalytic histidine could be a critical difference between glucoside and glucose ester formation. At the physiological pH for plants (~7.2 in cytoplasm), both SAG and SGE are produced in measurable quantities in *A. thaliana* cellular extracts (Fig. 1d). However, the production of SGE is ~3-fold greater at a pH lower than 6.5, and remains constant between pH = 7.0–8.0. SAG production is not detected at a pH lower than 6.0, increases from pH 6.0–7.0, and stays constant between pH = 7.0–8.5 (Fig. 1d). The pH dependence of the enzyme activity for the purified recombinant proteins (Fig. 1e and f) recapitulates the product specificity observed at pH 7.0 (Fig. 1b and c) for all other pH values and shows that SAG formation by UGT74F1 increases with the pH, for pH higher than 6.5, while SGE formation by UGT74F2 increases with decreasing pH for pH lower than 6.5. Finally, comparison of pH-dependent product formation between purified recombinant UGT74F1 and UGT74F2 and *A. thaliana* cellular extracts (Fig. 1d–f), suggests that UGT74F2 is expressed at higher levels than UGT74F1 (~10-fold) in *A. thaliana*.

### Crystallization of UGT74F2 and identification of SA binding site

UGT74F2, in conditions lacking UDP or UDP-glucose (for example, apo-enzyme or protein with SA or 2-BA), did not crystallize. Nevertheless, in the presence of UDP (as UDP or UDP-glucose; see below), UGT74F2 crystallized in complex with different ligands (Table 1). Soaking of SA or UDP-glucose into crystals resulted in disordered and low-resolution (less than 5 Å) crystal diffraction. Over 300 crystals were screened and, generally, co-crystallization of the protein with UDP/UDP-glucose and SA produced a smaller fraction of well-diffracting crystals (diffraction resolution up to 2.5 Å, ~5%) than the protein co-crystallized with UDP/UDP-glucose and 2-BA (~60%). Attempts to determine phasing information by molecular replacement with homologous structures failed, therefore we produced Seleno-Methione substituted UGT74F2 crystals and used Multiwavelength Anomalous Dispersion (MAD) method for structure solution. Irrespective of the ligation, UGT74F2 crystals had the same space group, with two monomers in the asymmetric unit, and similar cell parameters (Table 1). Both chains are very similar (all-atom RMSD 1.4 Å^2^) with two of the exposed loops showing different conformations (residues 47–55 and 382–392); however the catalytic site and core structure are virtually identical between the two chains (Supplementary Fig. S1).

**Figure 2:**
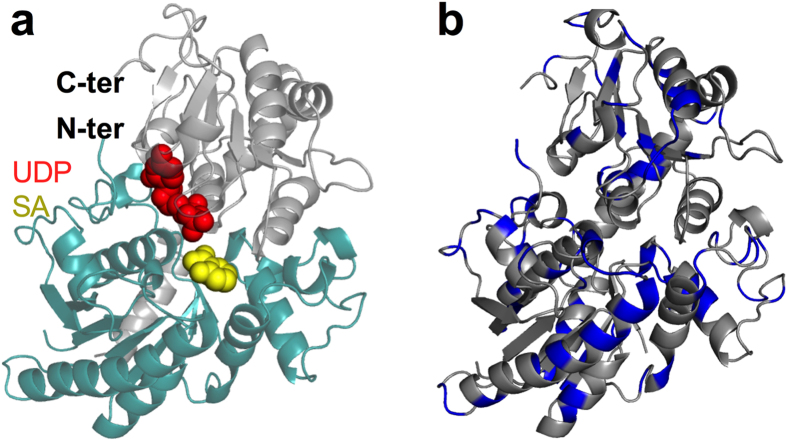
Overview of UGT74F2 crystal structure. (**a**) A chain of UGT74F2 in complex with UDP (red spheres) and SA (yellow spheres). N-terminal domain (residues 4 to 245) is colored teal with the C-terminal domain in grey. (**b**) Amino acid differences between UGT74F1 and UGT74F2 marked as blue on the UGT74F2 structure (see also Supplementary Fig. S3).

UGT74F2 exhibits the GT-B fold with ligands bound in the cleft between the two domains –the N-terminal domain is comprised of residues 1–245, while 246–449 make up the C-terminal domain (Fig. 2a). UDP and SA interact primarily with residues in the C- and N-terminal domain, respectively (Fig. 2a). There is an aqueous cavity in the SA binding domain (Supplementary Fig. S2a).

Despite systematic screening, UGT74F1 has not crystallized. Given the protein sequence identity to UGT74F2 (77%), we constructed a homology model of UGT74F1 based on the UGT74F2 crystal structure. The residues that are not conserved between the enzymes are spread throughout the structure (Fig. 2b), with a single conservative substitution in the ligand binding site – residue 15 is a serine in UGT74F1, but a threonine in UGT74F2 (Supplementary Fig. S3).

We compared UGT74F2 to previously crystallized homologs (Supplementary Table S1) by superposing the proteins at the UDP-glucose binding site (residues 332–362 in UGT74F2). Despite low sequence conservation (26–30% sequence identity, Supplementary Table S1), all of the UGT protein structures display the same secondary structure architecture (Supplementary Figs S2 and S3). The C-terminal domain includes the nucleotide binding site and is very similar (Supplementary Fig. S2b). On the other hand, the N-terminal domain containing the acceptor binding site exhibits shifts and rotations, reflected in all-atom RMSD of 5–10 Å^2^ between homologs and UGT74F2 (Supplementary Table S1 and Supplementary Fig. S2c).

**Figure 3:**
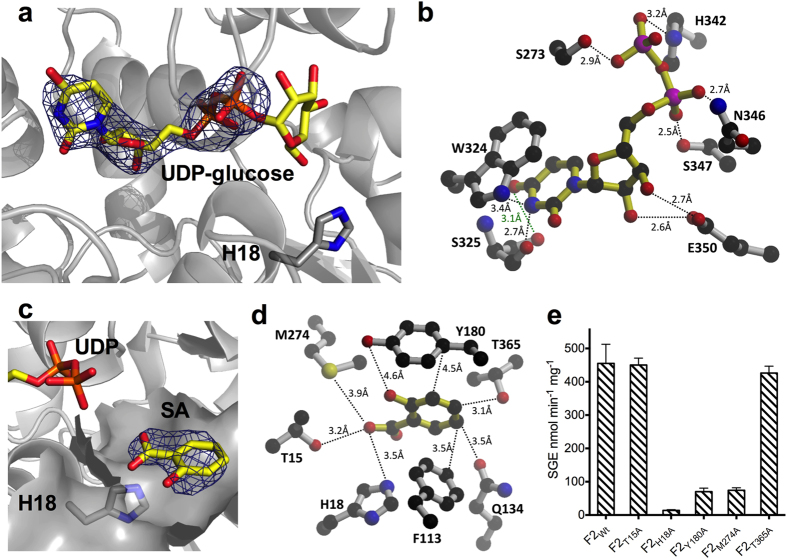
Ligand binding to UGT74F2. (**a**) Omit electron density for UDP (1σ, blue mesh) shows clear density for UDP, but not for terminal glucose (UDP-glucose in yellow sticks). (**b**) Key residues interacting with UDP and potential hydrogen bonds. (**c**) Omit electron density (1σ, blue mesh) for SA. (**d**) Residues interacting with SA (for clarity we omitted residues Met 183, Val 184 and Trp 364). (**e**) SGE production by UGT74F2 mutants of the SA-binding site at 3 min in standard assay conditions. Error bars represent standard deviation from three measurements.

Attempts to co-crystallize UGT74F2 and UDP-glucose yielded structures with strong electron density for UDP and weak or missing electron density for the glucose moiety in the omit map (Fig. 3a). This has been observed with other UGT structures, likely arising from hydrolysis of the glucose phosphate bond during crystallization. UDP forms interactions with a conserved binding site composed of Ser 273, Trp 324, His 342, Asn 346, Ser 347, and Glu 350 among others (Fig. 3b). Specifically, the indole ring of Trp 324 stacks with the pyrimidine ring of UDP; Glu 350 carboxylate makes hydrogen bond interactions with the two hydroxyl groups of the ribosyl moiety; and UDP phosphoryl groups interact with the side chains of Ser 273, His 342, Ser 347 and Asn 346. These residues are conserved in UGT74F1, UGT74F2 and other UGTs with known crystal structures (Supplementary Fig. S3).

**Figure 4:**
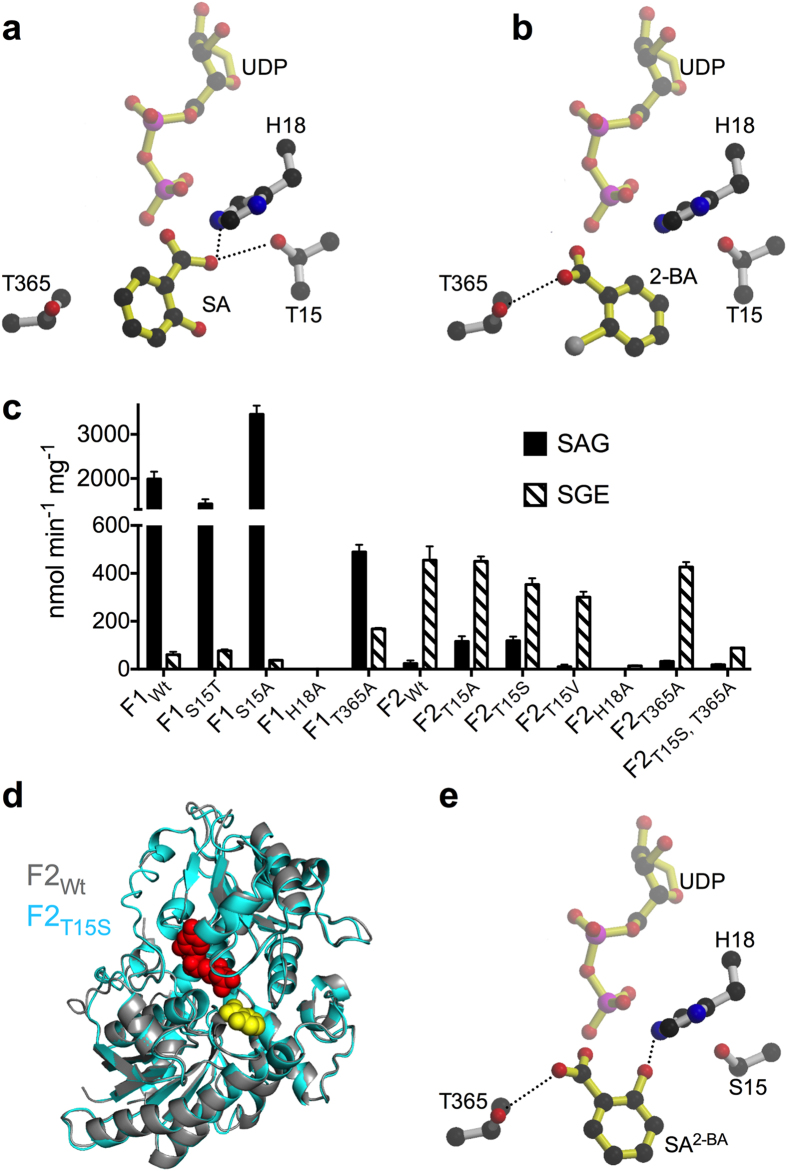
SA binding to UGT74F1 and UGT74F2. (**a**) In wild-type UGT74F2 UDP/SA complex structure, the carboxyl group of SA interacts with Thr 15 and His 18, and faces UDP-glucose binding site. (**b**) In UGT74F2 wild-type and T15S UDP/2-BA complex structures 2-BA binds with the carboxyl group close to Thr 365. (**c**) SAG and SGE production of mutant proteins at 3 min in standard assay conditions compared to wild-type UGT74F1 and UGT74F2. Error bars represent standard deviation from three measurements. (**d**) Overlay of UGT74F2 wild-type (gray) and UGT74F2_T15S_ (cyan) structures. (**e**) SA binding to the active site of UGT74F2_T15S_ (or UGT74F1), modeled on the basis of 2-BA (SA^2–BA^, see **b**). The carboxyl group of modeled SA interacts with Thr 365, while SA hydroxyl interacts with His 18 and faces UDP. Dashed lines show hydrogen bond interactions.

To identify the binding pocket of SA, UGT74F2 was co-crystallized with UDP and SA or SA analogue 2-bromobenzoic acid (2-BA). In the co-crystal of UGT74F2 with UDP and SA, the SA omit map shows clear density for a small molecule within a pocket in the acceptor binding domain (Fig. 3c). Two different conformations of SA were modeled in the omit electron density (Supplementary Fig. S4a and b). Analysis of the crystallographic thermal parameters (B factors) of SA and surrounding protein residues (Supplementary Table S2) support the SA conformation shown in Fig. 3c. Tyr 13, Thr 15, His 18, Phe 113, Gln 134, Tyr 180, Met 183, Met 274, Val 184, Trp 364 and Thr 365 delineate the SA binding pocket. The residues that form the binding pocket are similar, but not identical between UGT74F2 and UGT74F1, and with the exception of His 18, are not conserved in other UGTs (Supplementary Fig. S3). Van der Waals interactions between the benzoic ring of SA and Phe 133, Tyr 180, Met 183, Met 274, Trp 364, and Thr 365 constrain the approximate location of SA, while the carboxylate group of SA interacts through hydrogen bonds with His 18 and Thr 15 (Fig. 3d). To verify the presence of a single SA in the binding domain, we co-crystallized UGT74F2 with the SA analog 2-BA. Collection of an anomalous X-ray diffraction set showed a single anomalous peak in the SA binding pocket (Supplementary Fig. S5). While there is partial overlap between 2-BA and SA binding sites, the location of the carboxyl group of 2-BA differs from that of SA and is within hydrogen bond interaction from Thr 365 (Fig. 4a and b).

The SA binding site was validated by activity assay of several mutant proteins where SA binding residues were mutated to alanine (Fig. 3e). As with other UGTs, the conserved catalytic His 18 is crucial for activity; UGT74F2_H18A_ had no activity. Tyr 180 and Met 274 are involved in either SA binding or catalysis, as UGT74F2_Y180A_ and UGT74F2_M274A_ catalyze the formation of reduced quantities of SGE. Given the position of Tyr 180 and Met 274 far from the catalytic site (>10 Å from His 18), these residues most likely contribute to SA binding and orientation and are not directly involved in catalysis. Mutation of Thr 15 and Thr 365 to alanine yielded mutant enzymes that are active and produce similar quantities of SGE with wild-type, suggesting that the interactions of Thr 15 and Thr 365 with SA are not essential for SGE formation.

### Threonine 15 and 365 are important for UGT74F2 and UGT74F1 specificity, respectively

Within the SA binding site, all residues are identical between UGT74F1 and UGT74F2 with the exception of position 15, which is a serine in UGT74F1 but threonine in UGT74F2 (Supplementary Fig. S3). To test the contribution of this minor substitution to enzyme specificity, we expressed and purified several mutants. UGT74F2_T15V_ has reduced specific activity for SGE formation compared to wild-type UGT74F2 (by ~33%), but is still specific for SGE as it forms negligible SAG (Fig. 4c). In contrast, UGT74F2_T15A_ and UGT74F2_T15S_ form both SGE and SAG (Fig. 4c); UGT74F2_T15A_ has wild-type activity for SGE formation, better than UGT74F2_T15S_, and both mutants form about 5-fold more SAG than the wild-type. Mutation of position 15 in UGT74F1 does not affect product specificity, as UGT74F1_S15T_ forms only SAG, though with reduced activity compared to UGT74F1 wild-type (Fig. 4c). Interestingly, UGT74F1_S15A_ has a 70% increase in SAG activity, compared to the wild-type (Fig. 4c). Thus the trend of increased SAG formation with decreased size of the side-chain in position 15 (i.e. Ala > Ser > Thr) is shared by both UGT74F1 and UGT74F2.

We crystalized UGT74F2_T15S_ and UGT74F2_T15A_ (Supplementary Fig. S6) in complex with UDP and SA or UDP and 2-BA. As the findings with both mutants were identical, we will present further the structures of UGT74F2_T15S_. The UDP and SA complex structure of this mutant is very similar to that of the wild-type enzyme, with an overall RMSD of 1.4 Å^2^ (Fig. 4d). In contrast to the wild type enzyme, the omit map for SA in the binding pocket of UGT74F2_T15S_ does not have clear electron density for the 2-hydroxyl of SA, despite the higher resolution of the latter structure (Supplementary Fig. S7, Table 1). Interestingly, for the UGT74F2_T15S_ in complex with UDP and 2-BA, the electron density for 2-BA and in particular for its carboxylate group is stronger and better defined than in the analogous complex of the wild-type protein (Supplementary Fig. S4c and d, Supplementary Table S2). Given the electron density ambiguity in how SA is positioned (Supplementary Fig. S7) and the mutation effect on enzyme activity for residues 15, 18 and 365 (Fig. 4c), on the basis of 2-BA binding to UGT74F2 wild-type and T15S, an alternative orientation of SA in the acceptor site of UGT74F2_T15S_ was modeled (SA^2–BA^, Fig. 4e); this conformation places the hydroxyl group of SA in the same position as the crystallographically observed carboxylate ion and the carboxylate ion of SA in the same position as the carboxylate of 2-BA (Fig. 4b). SA^2–BA^ in UGT74F2_T15S_ would interact in the acceptor site similarly as in wild-type UGT74F2 (Fig. 4a) with two major differences. First, the hydroxyl group of SA^2–BA^ is within hydrogen bond distance from the catalytic His 18, while Ser 15 is too far for interaction. Second, Thr 365 forms a hydrogen bond with the SA carboxyl. This alternate binding of SA would be consistent with formation of the SAG product, which is produced by UGT74F2_T15S_ and UGT74F1 (Fig. 4c).

To investigate if the predicted interaction between Thr 365 and SA is important in UGT74F1, we mutated Thr 365 to alanine in UGT74F1; the same mutation in UGT74F2 did not affect SGE formation (Fig. 3e). Compared to wild-type UGT74F1, UGT74F1_T365A_ exhibited 75% decreased activity for SAG, but 3-fold increase in SGE formation activity (Fig. 4c), though still well below SAG production. As for UGT74F2, while each individual mutation (T365A or T15S) did not significantly affect the activity, compared to the wild-type UGT74F2, the double mutant UGT74F2_T15S, T365A_ displayed an 85% decrease in activity for SGE production (Fig. 4c).

### Proposed catalytic mechanisms

**Figure 5:**
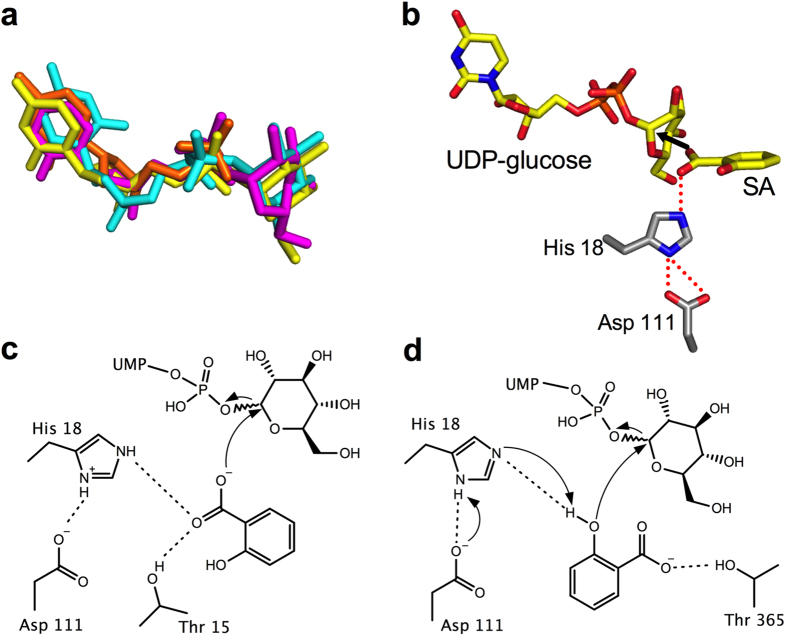
Proposed catalytic mechanism for SA glucose conjugate formation. (**a**) Modeled UDP-glucose molecules. Crystallographically observed UDP shown in orange, UDP-2-fluoro-glucose from PDB 2c1z shown in yellow, UDP-glucose modeled by Molecular Operating Environment is in cyan, and UDP-glucose modeled from incomplete electron density is shown in magenta. (**b**) View of proposed catalytic site for UGT74F2 showing modeled UDP-glucose, SA and the dyad His 18 - Asp 111. (**c**) View of catalytic site to form SGE, based on (**b**) and Fig. 4a: SA carboxyl oriented towards UDP-glucose by interactions with His 18 and Thr 15, performs an S_N_2 attack by the carboxylate oxygen to the anomeric carbon of UDP-glucose. (**d**) View of catalytic site for SAG formation, based on Fig. 4e: SA hydroxyl deprotonated by His 18 - Asp 111 catalytic dyad; S_N_2 attack by the adjacent aromatic hydroxyl to the anomeric carbon results in the formation of SAG, and the proton is released from His 18 to regenerate the active site.

To approximate the glucose position in UGT74F2, we utilized a combination of three models (Fig. 5a). First, we placed UDP-glucose into the observed poor electron density of the UDP-glucose co-crystal. Second, we modeled UDP-glucose on the basis of the non-transferable UDP-glucose analog UDP-2-fluoro-glucose from VvGT1 crystal structure (PDB 2c1z)^26^. Third, with Molecular Operating Environment (MOE, http://www.chemcomp.com), we docked UDP-glucose into the catalytic site of our UGT74F2 structure with UDP removed. These three models agree that UDP-glucose binds in a similar manner to UDP in the conserved nucleotide binding site, with the glucose moiety positioned towards the SA binding site (Fig. 5a and b). Based on the modeled UDP-glucose, crystallographically observed SA and mutagenesis studies, we propose the minimal active site for UGT74F2 (Fig. 5b). The conserved catalytic His 18-Asp 111 dyad is below SA; there is a hydrogen bond from His 18 to the carboxylate group of SA, which is positioned 2.6 Å from the anomeric carbon of UDP-glucose.

Based on the observed SA and modeled UDP-glucose, we propose a S_N_2 reaction for SGE formation by UGT74F2 (Fig. 5c). SA carboxyl is positioned towards the anomeric carbon of UDP-glucose by hydrogen bond interactions with His 18 and Thr 15 (Fig. 4a and c). A branched small amino acid in position 15 (Thr or Val) is probably necessary to restrict the binding mode of SA (Fig. 4c). Given the pH dependence of SGE formation (Fig. 1f) and the critical role of His 18 in UGT74F2 activity (Fig. 3e), His 18 is probably protonated and forms hydrogen bond interactions both with Asp 111 and one of the oxygens of the SA carboxylate group. The other carboxylate oxygen is now an oxyanion with little resonance stabilization; it attacks the anomeric carbon of UDP-glucose, which is 2.6 Å away. This mechanism would result in an inversion of stereochemistry at the anomeric carbon of glucose (α to β), which has been previously observed in SGE formed by cell extracts^16^.

SAG formed in *A. thaliana* cell extracts have β stereochemistry at the glucose moiety^16^, which makes UGT74F1 an inverting enzyme, consistent with previously studied glucoside forming enzymes^26^. Based on alternatively bound SA (SA^2–BA^, Fig. 4e) to the enzyme active site, we propose that the active site of UGT74F1 is composed of His 18, Asp 111, and Thr 365 (Figs 4e and 5d). The hydroxyl of SA would be deprotonated by His 18 acting as a catalytic base, and then performs a nucleophilic attack to the anomeric carbon of UDP-glucose, generating SAG, again with inversion of configuration. While we tried to confirm the involvement of Asp 111 in the catalysis, our efforts to purify the mutants for this position either in UGT74F1 or UGT74F2 have been unsuccessful.

## Discussion

SA is a signaling hormone involved in the immune response of plants. The glucosylated form of SA determines how it will be transported or stored in the plant cell. UGT74F1 and UGT74F2 are the enzymes responsible for the *in vivo* glucosylation of SA in *A. thaliana*. Homologs of these proteins are present in many other plant species, including fruiting trees, citrus trees and the *Brassica* genus, suggesting that these enzymes and their products work together in plant immune responses.

UGT74F1 and UGT74F2 share 77% identity at the sequence level and utilize the same substrates, yet they create different products. In UGT74F1, glucose is transferred to the hydroxyl of SA, creating a glucoside; in UGT74F2, glucose is transferred to the carboxylate group of SA to create a glucose ester. We expressed, purified and biochemically characterized recombinant UGT74F1 and UGT74F2 and solved the crystal structure of UGT74F2 to determine the molecular determinants of product specificity in these enzymes.

For UGT74F2, we identified four mutations that negatively impacted enzyme function. Mutation of His 18 to Ala abolished activity, consistent with the central role of His 18 in catalysis. Mutation of Tyr 180 to Ala reduced activity by ~80%, even though the side chain is far from the active site, so it is unlikely that Tyr 180 is involved in catalysis. More likely, Tyr 180 is important for ligand recognition or binding; other studies have found that residues in this region are important for enzyme function^29^,^30^. Additionally, Met 274 may be important for catalysis; its mutation to alanine decreased activity by ~80% compared to the wild-type enzyme. The contribution of this position to catalysis and binding is unknown, but given its proximity to both SA and the modeled glucose moiety of UDP-glucose, it could be crucial for orientation of the SA.

We identified two residues that are important for UGT74F1 and UGT74F2 product specificity. The only amino acid substitution in the SA binding site is a serine (UGT74F1)/threonine (UGT74F2) substitution at position 15. Relative to the wild-type enzyme, mutation of residue 15 to serine or alanine in UGT74F2 did not lessen the specific activity for SGE formation, but did increase the formation of SAG. Mutation of Thr 15 to valine (another branched amino acid) did not affect product specificity. Mutation of Ser 15 to threonine in UGT74F1 did not affect product specificity but mutation to alanine increased significantly SAG formation. Altogether, these data suggest that the structure of residue 15 is important for SAG formation in both UGT74F1 and UGT74F2: the smaller the side-chain, the higher SAG formation (with Ala > Ser > Thr). Perhaps the presence of a branched amino acid at residue 15 constrains SA binding in a particular conformation and functions in organizing the binding pocket. Thr 365 forms part of the SA binding site in UGT74F2, but mutation to alanine did not affect specific activity or product specificity. Based on modeling of SA binding to UGT74F1 (Fig. 4e), we predicted that Thr 365 could be part of the active site for SAG formation. Indeed mutation of Thr 365 to alanine in UGT74F1 decreased SAG activity by 75% and increased SGE activity by 300%, suggesting an important role of this residue in catalysis and substrate recognition. Interestingly, the residues implicated in product discrimination are two threonines proposed to each interact with the carboxyl group of SA, which in turn is bound in distinct conformations in UGT74F1 and UGT74F2. Therefore, we propose that SA binds to UGT74F2 primarily in the conformation described for the UDP/SA complex (i.e. carboxyl group of SA interacting with His 18 and Thr 15; Fig. 4a) but binds to UGT74F1 primarily in the conformation modeled on the basis of 2-BA in the 2-BA/UDP complexes of UGT74F2 wild-type or T15S (i.e. SA hydroxyl interacting with His 18 and SA carboxyl interacting with Thr 365, Fig. 4e). This explains why UGT74F1_S15T_ and UGT74F2_T365A_ behave like wild-type enzymes (no carboxyl group from SA to interact with) while UGT74F2_T15S_ and UGT74F1_T365A_ impact product specificity (both mutations affect interaction with SA carboxyl); the latter mutants can accommodate both binding modes of SA. Therefore, impairing the recognition of SA carboxyl group in each enzyme decreases the product specificity.

Distinct binding modes of SA for UGT74F1 and UGT74F2 are also supported by the pH dependence of the enzyme activity. Although pH values lower than 6.5 exhibit increased SGE formation in UGT74F2, they have no effect on SGE formation by UGT74F1 and, vice versa, at pH values higher than 6.5 SAG formation by UGT74F1 increases but is unchanged in UGT74F2. As the catalytic His 18 is conserved in both enzymes, the independence of pH for product specificity in both enzymes is consistent with each enzyme preferring a certain binding mode for SA. Given the amazing active site conservation between UGT74F1 and UGT74F2 it is unclear why a distinct conformation of SA is strongly favored in a particular enzyme. It is possible that slight perturbations in the loops that contribute residues to the active site influence the glucosyl acceptor site organization so that a certain SA binding mode is preferred.

Based on the structures of co-crystallized ligands and modeled UDP-glucose into the UGT74F2, we propose that SGE is formed by S_N_2 attack from the SA carboxylate group. His 18 plays a critical role in catalysis by removing resonance stabilization in the carboxylate group and activating the oxyanion for attack. For UGT74F1, we propose that SA binds in a different orientation with the hydroxyl pointed towards UDP-glucose, and that S_N_2 attack from the deprotonated hydroxyl causes the formation of SAG. The His 18 - Asp 111 dyad deprotonates SA hydroxyl, with His 18 acting as a catalytic base.

The proposed mechanisms rely on activation of an oxygen-containing group, but a crucial difference between these reactions is the beginning state of the oxygen. For UGT74F2, the carboxylate group of SA is already ionized for attack; in UGT74F1, the hydroxyl group of SA must be deprotonated before a nucleophilic attack is possible. These functional groups have different pK_a_ values (~3 vs. ~13 for the carboxyl and hydroxyl groups, respectively). The pH dependence for formation of both SAG and SGE, with SGE production favored at pH < 6.5 and SAG formation favored at pH > 6.5, further supports the proposed catalytic mechanisms. Formation of SGE requires activation of an already ionized carboxylate group, while SAG formation depends upon deprotonation of the SA hydroxyl. The proposed hydrogen bonding in UGT74F2 requires that His 18 be the hydrogen bond donor for both bonds, so it must be protonated. The pKa of histidine residues is ~6.6^35^, so His 18 would be protonated below pH ~6.5, consistent with the observed pH dependence. To form SAG, His 18 deprotonates SA hydroxyl, and thus must be deprotonated before the reaction begins. This mechanism is consistent with the observed increase of SAG production above pH 6.5.

In this work, we report the first crystal structure of a UGT that preferentially catalyzes the formation of glucose ester products and propose a mechanism for glucose ester formation. We also identified a single residue that is important for product specificity by UGT74 enzymes. As has been proposed for other UGTs^36^, we theorize that the specificity of UGT74F1 and UGT74F2 is primarily determined by orienting SA in the binding site. Unlike UGTs that act on flavonoids, however, UGT74F1 and UGT74F2 catalyze chemically distinct reactions and may be able to harness those distinctions to assist in specificity. These studies should inform future experiments exploring different product formation by UGTs.

## Materials and Methods

### UGT74F1 and UGT74F2 expression in *Escherichia coli*

Codon optimized genes for UGT74F1 and UGT74F2 were ordered from Genscript and cloned into pET15(+)b with a thrombin-cleavable N-terminal hexa-histidine tag. Plasmids were transformed into *E. coli* strain C41(DE3)^37^ and selected on Luria Broth (LB) agar plates with 150 μg/ml carbenicillin. Pilot cultures of LB containing 150 μg/ml ampicillin were inoculated and grown overnight at 37 °C with shaking. For protein expression, 7 L fermenter of LB with 150 μg/ml ampicillin was inoculated from the pilot culture and grown at 37 °C until OD_600_ = 0.8; culture was induced with the addition of 0.4 mM IPTG and growth continued for 4 hours at 37 °C. For selenomethionine labeled protein expression, cells were grown in M9 media with amino acid supplements^38^. Cells were harvested by centrifugation (5,000 × g, 10 minutes, 23 °C) and frozen at −20 °C until lysis. To generate DNA for mutant proteins, site-directed mutagenesis was performed on the pET15(+) plasmid constructs of wild-type proteins and verified by DNA sequencing.

### Protein Purification

The thawed cell pellet was resuspended in 120 mL of buffer A [50 mM sodium phosphate (NaPi) (pH 7.5), 5% (v/v) glycerol, 200 mM sodium chloride (NaCl)], with 2 mM phenymethylsulfonylfluoride, 2 mM magnesium chloride, lysozyme (30 mg) and DNAse (10 mg) at 4 °C, and disrupted by sonication (Branson Ultrasonic). The cell lysate was clarified by centrifugation at 16,000 × g and 4 °C for 1 hour, and the supernatant was loaded onto the Ni-NTA resin (EMD Millipore) and washed with buffer containing 50 mM NaPi (pH 7.5), 500 mM NaCl, 5–20 mM imidazole, 5% (v/v) glycerol. Protein was eluted with buffer A, containing 300 mM imidazole, then concentrated and digested overnight at 4 °C with thrombin (Biopharma, 10 unit/mg protein). His-tag free protein was loaded again on Ni-NTA resin and the pass-through was collected and concentrated to 10–20 mg/ml. For final purification, proteins were separated on a Superdex200 column (GE Healthscience) in crystallization buffer [20 mM TrisHCl (pH 8), 100 mM NaCl], yielding greater than 95% pure samples, as judged by SDS-PAGE. Protein was concentrated to 10–20 mg/ml, flash-frozen in liquid nitrogen, and frozen at −80 °C until use. Mutant proteins were expressed and purified in the same manner as wild-type proteins with no modifications.

### Salicylic acid conjugation activity assays

The assay conditions for the purified proteins were as described^15^ except that [7-^14^C]SA (specific activity 1.5 μCi μmol^−1^; PerkinElmer, Boston, MA) was substituted for the unlabeled SA. Assays with *A. thaliana* cell lysate were as described previously^16^. HPLC separations of the glucose conjugates were performed with a 150 × 4.6 mm Alltima HP C18 5μ column (Grace Discovery Sciences, Deerfield, IL, USA) that was eluted at a flow rate of 1 ml min^−1^ with a linear gradient from 95% acetic acid (1%) and 5% methanol to 20% acetic acid (1%) and 80% methanol in 20 min. Eluant from the column was diverted to a fraction collector and the radioactivity in 0.5-ml fractions was determined through liquid scintillation counting. SA, SAG and SGE were identified as previously described^16^.

### Protein crystallization and structural determination

Crystallization by hanging-drop vapor diffusion was set up by combining 1 μL of 10 mg/mL purified UGT74F2 or UGT74F2_T15S_ or UGT74F2_T15A_ in the presence of 2 mM UDP-glucose (or UDP) with or without 5 mM SA (or 2-BA) with 1 μL of precipitant solution. Crystals appeared within 3–5 days, at room temperature, at 22–26%(w/v) PEG3350, 0.1 M MES (pH 5.5), 0.2 M ammonium acetate.

All x-ray diffraction data were collected at Beamline 23-ID-B, GM/CA-CAT at the Advanced Photon Source, Argonne National Laboratory, Lemont, IL. Initial phasing was obtained from crystals of selenium-methionine substituted UGT74F2 by multi-wavelength anomalous diffraction (λ1 = 0.9795, λ2 = 0.9641, λ3 = 1.0003) using the program ShelxC/D/E^39^. The tracing of amino acids was done with the program ARP/wARP^40^ and COOT^41^. The initially built model was subjected to molecular replacement to native data complexed with UDP, and all other crystal structures were solved with the program Phaser^42^. The model was built using COOT^41^ and refined with Phenix^43^ and Refmac^44^. RMSD between a crystal structure of UGT74F2 and other previously reported UGTs crystal structures were calculated with Superpose^42^. Figures were generated using Pymol (http://www.pymol.org), Molscript^45^ and Raster3D^46^.

### *In silico* ligand docking

*In silico* docking was performed in Molecular Operating Environment (MOE, http://www.chemcomp.com) with Amber10:EHT forcefield and R-field solvation. UDP-glucose docking into UGT74F2: A library of 55 possible conformations of UDP-glucose was generated with Conformation Search using the LowMode MD algorithm (no rigid-body, no fixed O-H bond lengths, unconstrained double-bond rotation). Before docking, UGT74F2 was prepared by protonation at pH 7.5 and then energy minimization. Ligand binding site was identified with SiteFinder and included residues Ser 273, Trp 324, His 342, Asn 346, Ser 347, Glu 350; dummy atoms were placed at this site for docking. UDP-glucose conformations were docked onto dummy atoms with Dock with all default parameters in Triangle Matcher retaining 100 poses with London dG scoring (estimates free energy of binding, based upon entropy changes, loss of flexibility of the ligand, hydrogen bond geometry, and desolvation of all atoms) and refined, retaining 30 poses using Alpha HB rescoring (with equal weights for hydrogen bonds and geometry of ligand-receptor fit). After docking, poses were sorted by ascending refinement score, and top 10 scored poses were screened for reasonable interactions with the protein based on physiochemical properties. To place alternatively bound SA in UGT74F2_T15S_, SA was manually placed in the binding site with 2-hydroxyl group in the small lobe in the ligand electron density and the benzene ring placed into the planar density. Because of the geometry of the surrounding residues, SA could only bind in one conformation in the binding site. This complex was imported into MOE, prepared by protonation at pH 7.5 and energy minimized.

## Additional Information

**How to cite this article**: George Thompson, A. M. *et al*. Differences in salicylic acid glucose conjugations by UGT74F1 and UGT74F2 from *Arabidopsis thaliana. Sci. Rep.* **7**, 46629; doi: 10.1038/srep46629 (2017).

**Publisher's note:** Springer Nature remains neutral with regard to jurisdictional claims in published maps and institutional affiliations.

## Supplementary Material

Supplementary Information

## Figures and Tables

**Table 1:** Crystallographic Data and Structure Determination Statistics for UGT74F2, UGT74F2_T15S_ and UGT74F2_T15A_ crystal structures.

	UGT74F2 UDP + SA	UGT74F2 SeMet UDP	UGT74F2 UDP + 2BA	UGT74F2_T15S_ UDP + SA	UGT74F2_T15A_ UDP + 2BA	UGT74F2_T15S_ UDP + 2BA
**Data collection**						
Space group	P2_1_2_1_2_1_	P2_1_2_1_2_1_	P2_1_2_1_2_1_	P2_1_2_1_2_1_	P2_1_2_1_2_1_	P2_1_2_1_2_1_
Cell dimensions						
*a, b, c* (Å)	65.70, 87.48, 164.25	66.10, 87.11, 162.95	65.15, 87.25, 162.12	65.18, 87.56, 163.02	65.12, 87.27, 162.67	65.22, 87.41, 162.83
a, b, g (°)	90, 90, 90	90, 90, 90	90, 90, 90	90, 90, 90	90, 90, 90	90, 90, 90
Resolution (Å)	2.56–50.00 (2.56–2.65)	2.20–50.00 (2.20–2.28)	2.00–50.00 (2.00–2.03)	2.00–100.00 (2.00–2.03)	2.00–50.00 (2.00–2.03)	1.80–50.00 (1.80–1.83)
*R*_sym_	0.086 (0.971)	0.115 (0.795)	0.086 (0.828)	0.056 (0.754)	0.054 (0.835)	0.075 (0.895)
*I*/sigma	33.2 (1.7)	34.3 (3.1)	38.3 (2.3)	48.6 (1.9)	29.8 (1.4)	19.2 (0.6)
Completeness (%)	99.3 (98.0)	100 (100)	100 (99.9)	99.7 (99.9)	100 (100)	99.4 (96.7)
Redundancy	6.9 (6.6)	12.0 (11.6)	9.1 (9.4)	6.3 (5.2)	6.8 (6.3)	5.2 (3.1)
MAD-Figure of merit (%)		50.6				
**Refinement**						
Resolution (Å)	2.56–43.8		2.0–37.5	2.0–50.9	2.0–44.0	1.8–42.2
No. reflections	30628		120959	63534	63039	90102
*R*_work_/*R*_free_	0.188/0.259		0.187/0.238	0.194/0.241	0.193/0.243	0.202/0.249
R.m.s. deviations						
Bond lengths (Å)	0.010		0.009	0.009	0.008	0.008
Bond angles (°)	1.191		1.042	1.031	0.918	0.914
PDB ID	5U6M		5U6S	5U6N	5V2K	5V2J

*R*_*sym*_ = Σ_j_ Σ_i_ |*I*_*ij*_ − < *I*_*j*_ > |/Σ_i_ Σ_*j*_ *I*_*ij*_, where i runs over multiple obervations of the same intensity, and j runs over all crystallographic unique intensities. R_factor_ = Σ ||*F*_*obs*_| − |*F*_*calc*_|| /Σ |*F*_*obs*_|. R_free_ was calculated with 5% of the reflections selected.
