# Supplementary material for: Differences in salicylic acid glucose conjugations by UGT74F1 and UGT74F2 from Arabidopsis thaliana
Source: Sci Rep. 2017 Apr 20;7:46629. doi: 10.1038/srep46629 (PMC5397973; doi:10.1038/srep46629)
Supplement: Supplementary Information [file srep46629-s1.pdf]

Supplementary Information

Differences in salicylic acid glucose conjugations by UGT74F1 and UGT74F2 from *Arabidopsis thaliana*

Alayna M. George Thompson[1], Cristina V. Iancu[1], Kenneth E. Neet[1], John V. Dean[2], Jun-yong Choe[1]

[1] Department of Biochemistry and Molecular Biology, Rosalind Franklin University of Medicine and Science, The Chicago Medical School, 3333 Green Bay Road, North Chicago, IL 60064, USA

[2] Department of Biological Sciences, DePaul University, 2325 N. Clifton Ave. Chicago, IL 60614, USA

Supplementary Table S1. Comparison of UGT74F2 UDP/SA complex structure to previously crystallized UGTs. Structures were aligned to UGT74F2 by overlaying the nucleotide binding domain (residues 332-362 in UGT74F2) with the program Superpose[42], and then all-atom RMSD were calculated.

Enzyme	Known *in vitro* major products	Ref.	Id.(%) to UGT74F1	Id. (%) to UGT74F2	PDB ID	Co-crystallized with	RMSD (Å)
UGT71 G1	Flavonoid O-glucoside [quercetin glucosides (3O' major product), geinistein-7-O-glucoside] Saponins (triterpene O-glucoside)	30	25.2	25.7%	2ACV	UDP	7.39
UGT72 B1	Monolignol O-glucoside Chloroaniline N-glucoside	31, 32	28.3	30.5%	2VG8	UDP, Tris	10.48
					2VCE	U2F, 2,4,5-trichlorophenol	10.39
					2VCH	UDP	10.37
UGT78 G1	Anthocyanidin 3-O-glucoside Flavonoid 3-O-glucoside	33	25.5	26.2	3HBJ	UDP	4.84
					3HBF	UDP + myricetin	4.89
UGT78 K6	Anthocyanidin 3-O-glucoside Flavonoid 3-O-glucoside	28	27.9	27.0	4REL	Kaempferol	5.09
					4WHM	UDP	5.05
UGT85 H2	Kaempferol 3-O-glucoside Biochanin A 7-O-glucoside	33, 34	30.8	30.4	2PQ6		6.86
GT1	Flavonoid 3-O-glucoside	26	27.1	26.6	2c1x	UDP	5.92
					2c1z	U2F, kaempferol	5.93
					2c9z	UDP, quercetin	6.11

Abbreviations: Ref., reference; Id., protein sequence identity

Supplementary Table S2. Average thermal factors (B parameters) of the carboxyl group in salicylic acid or in 2-bromobenzoic acid relative to those of side chain atoms for residues 15 (C_β) and 365 (C_β, C_γ and O_γ). SA has two conformations: SA and $SA^{alternate}$ (see Supplementary Fig. 4). Protein crystal structures used were: SA/UDP complexes for $UGT74F2_{wt}$ and $UGT74F2_{T15S}$ (PDB ID 5U6M, 5U6N), 2-BA/UDP complexes for $UGT74F2_{wt}$, $UGT74F2_{T15S}$ and $UGT74F2_{T15A}$ (PDB ID 5U6S, 5V2J, 5V2K). Ratio is [Average B for Ligand (A)] / [Average B for Residues 15 and 365 (B)]; a lower ratio indicates a higher occupancy for the ligand.

Protein complex	Average B for Ligand (A)	Average B for Residues 15 and 365 (B)	Ratio (=A/B)
SA in $UGT74F2_{wt}$	100	68	1.5
SA in $UGT74F2_{T15S}$	95	50	1.9
$SA^{alternate}$ in $UGT74F2_{wt}$	134	70	1.9
$SA^{alternate}$ in $UGT74F2_{T15S}$	119	51	2.3
2-BA in $UGT74F2_{wt}$	106	31	3.4
2-BA in $UGT74F2_{T15S}$	55	29	1.9
2-BA in $UGT74F2_{T15A}$	58	33	1.8

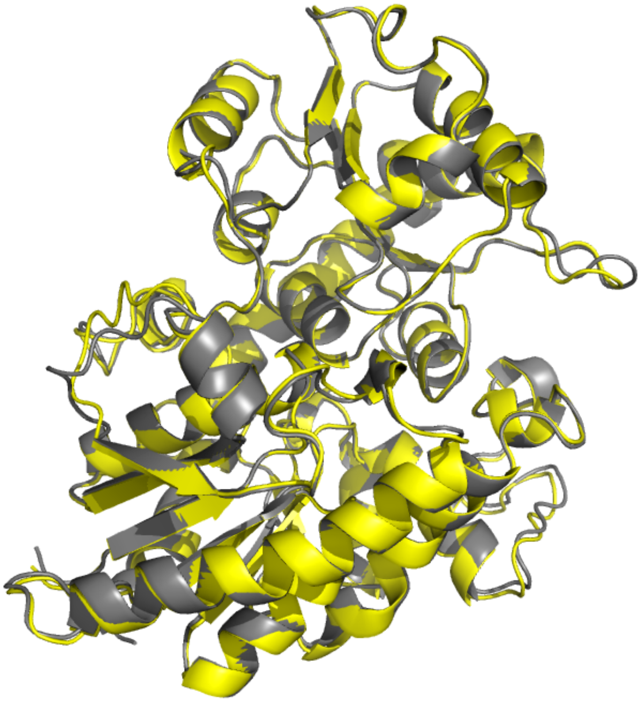

Supplementary Figure S1. A (gray) and B (yellow) chains of UGT74F2 in complex with UDP and SA.

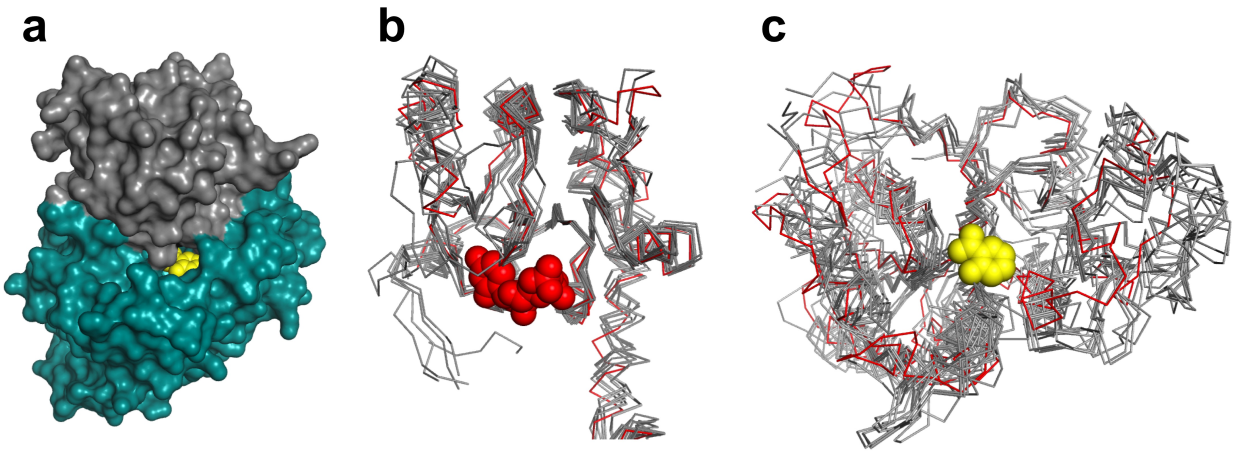

Supplementary Figure S2. Crystal structure of UGT74F2 in complex with UDP and SA - comparison to homologs. (**a**) Surface view of the crystal structure; SA bound to UGT74F2 is visible through an aqueous cavity. (**b**) UDP (red balls) and C-terminal UGT74F2 (red trace) compared to superposed homologs (grey traces). (**c**) SA (yellow balls) and N-terminal UGT74F2 (red trace) compared to superposed homologs (grey traces).

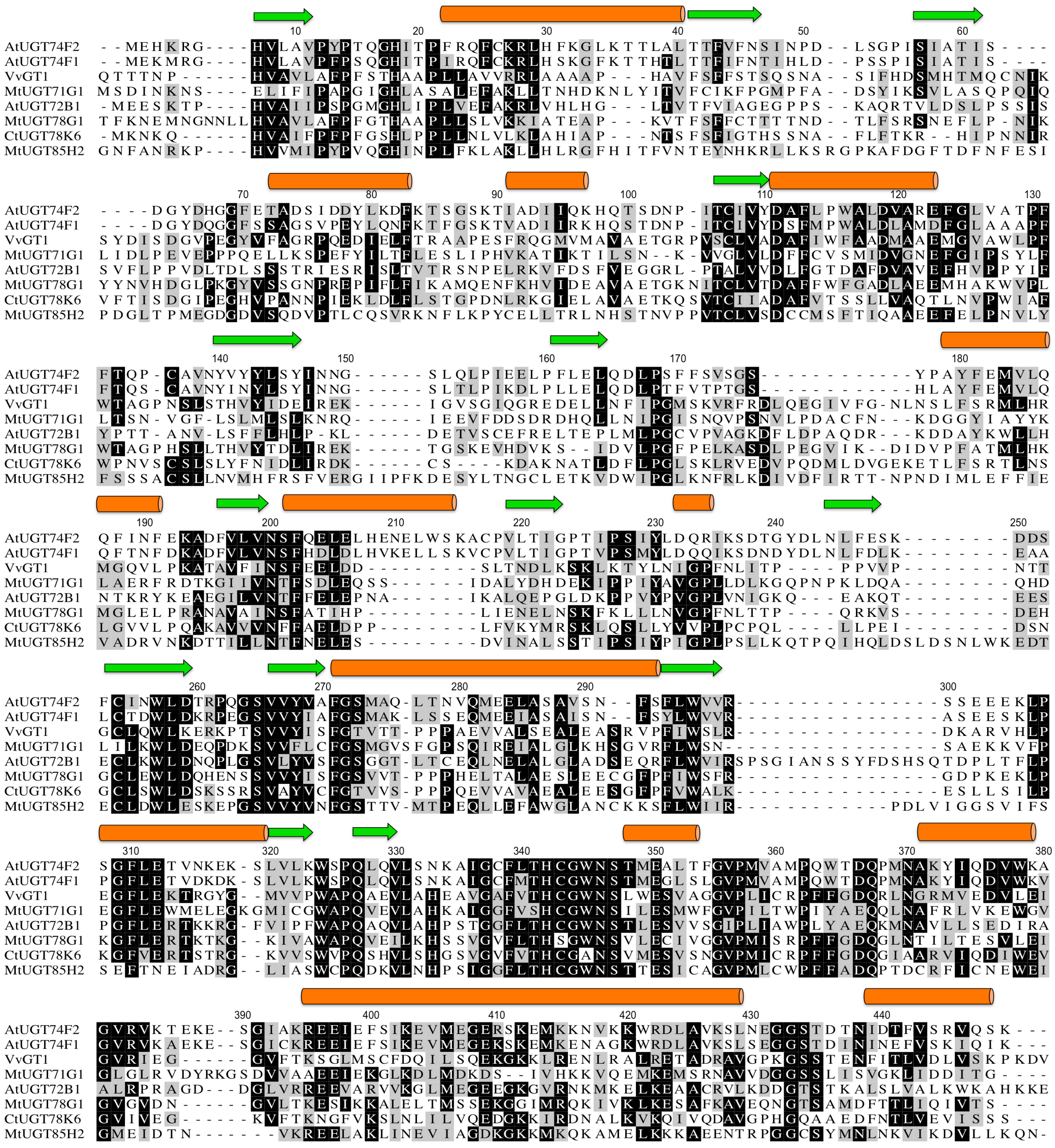

Supplementary Figure S3. Sequence alignment of UGT74F2 and UGTs with known crystal structures.

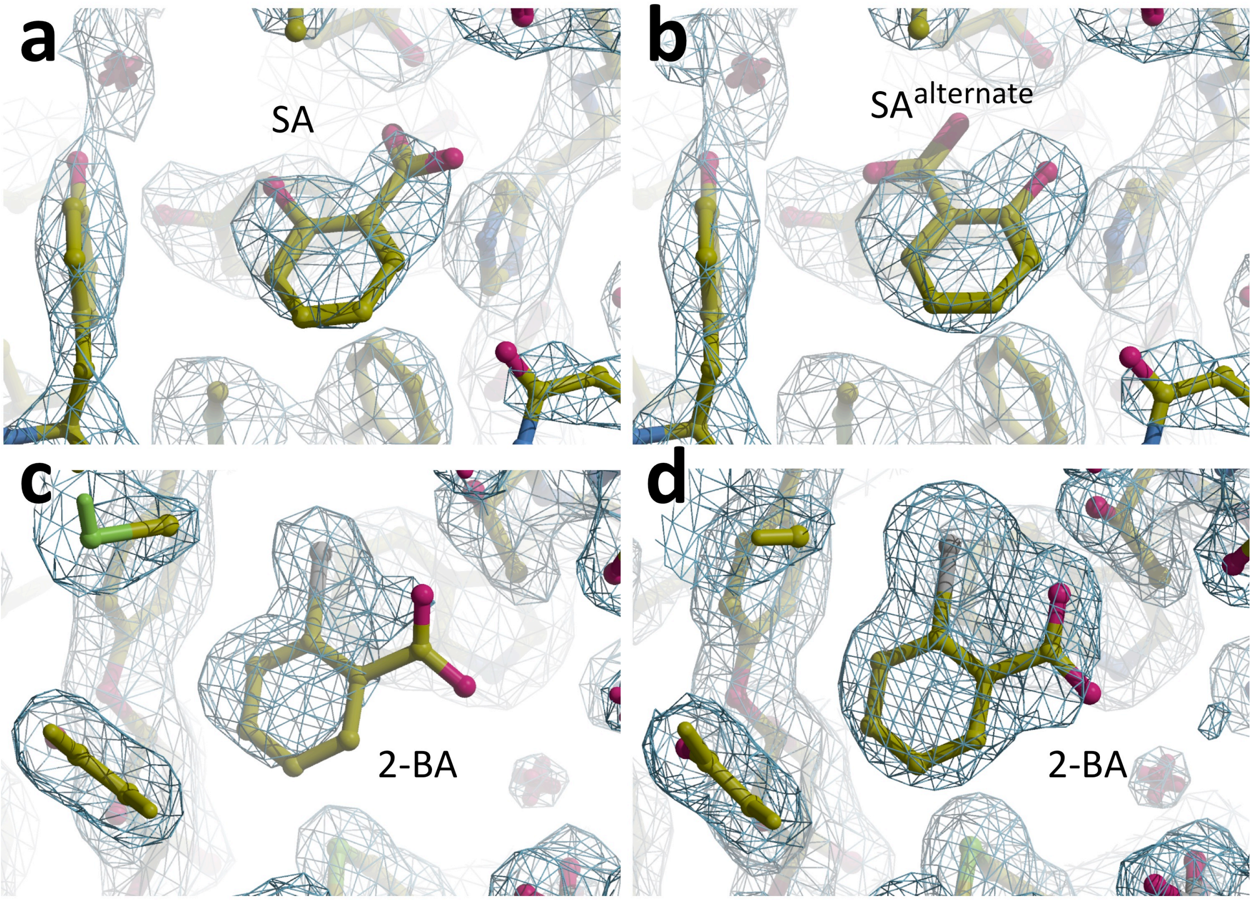

Supplementary Figure S4. Salicylic acid and 2-bromobenzoate in 2Fo-Fc electron density map (1.2 rmsd contour level). Two different conformations of salicylate in electron density map of UGT74F2 SA/UDP complex: the canonical conformation (SA in PDB ID 5U6M; **a**) and an alternate conformation (SA^{alternate}, **b**). Average thermal parameters analysis of these conformations (Supplementary Table 2) support SA conformation shown in (**a**) for the UGT74F2 SA/UDP complex. 2-bromobenzoate in wild-type (**c**) and T15S (**d**) mutant 2-BA/UDP complexes (PDB IDs 5U6S and 5V2J). Average thermal parameters analysis (Supplementary Table 2) shows that 2-BA has higher occupancy in the mutant relative to the wild-type.

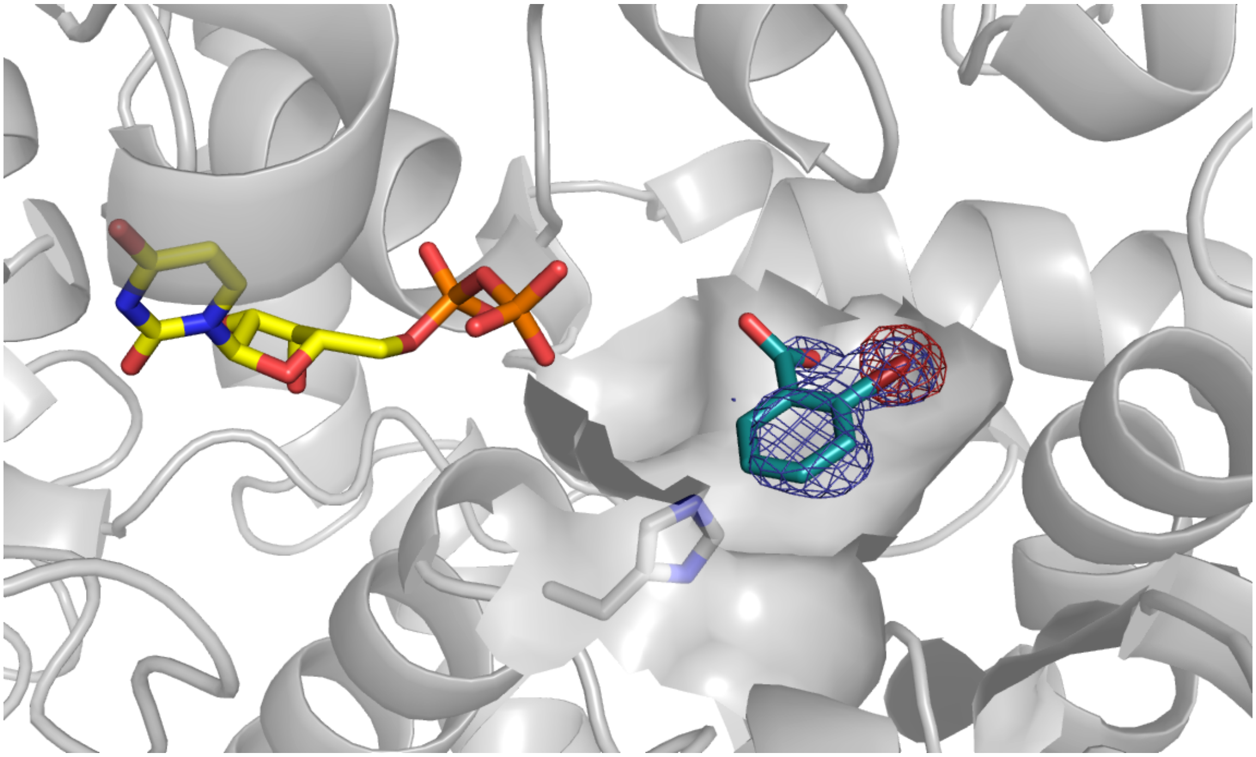

Supplementary Figure S5. 2-bromobenzoic acid (2-BA) in UGT74F2 acceptor binding site shown in omit map (1σ, blue mesh); the anomalous difference density map (4σ, red mesh) indicates the position of Br in 2-BA.

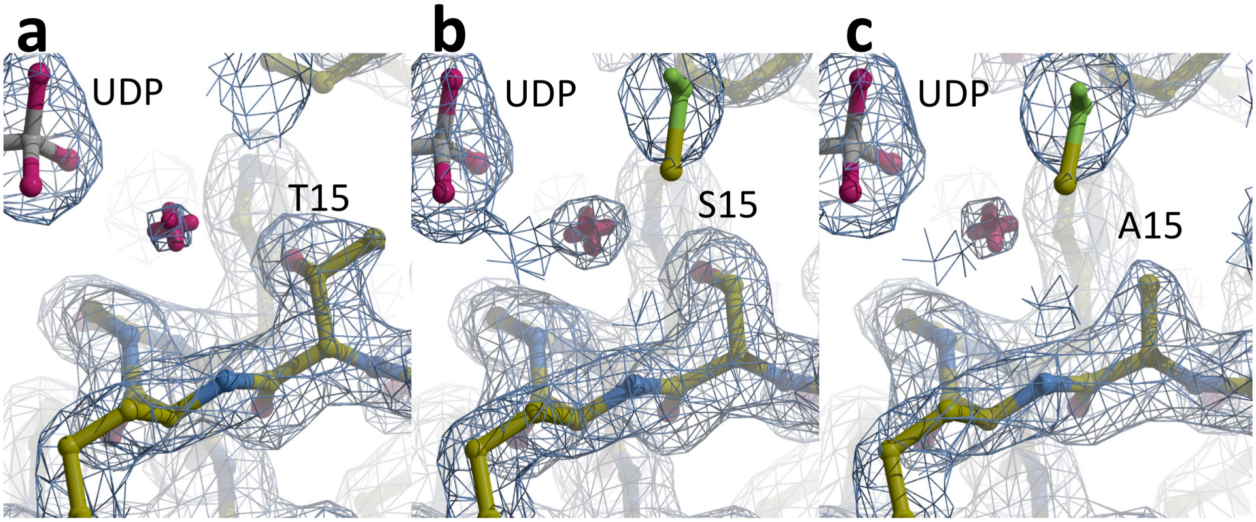

Supplementary Figure S6. 2Fo-Fc electron density map (1.5 rmsd contour level) of UGT74F2 amino acid residue 15 in wild type (**a**), T15S (**b**), and T15A (**c**) UDP/2-BA complex structures.

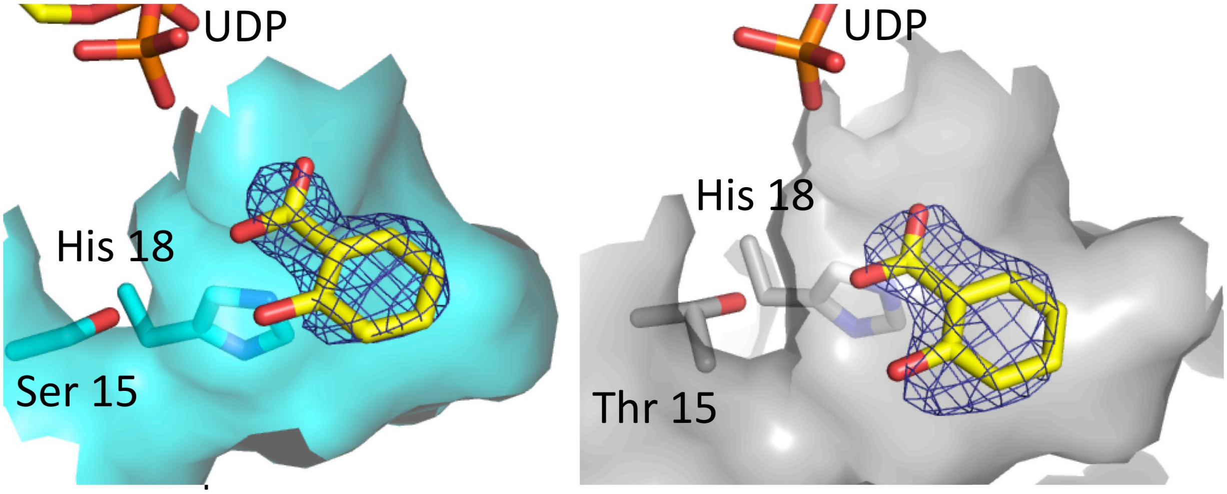

Supplementary Figure S7. Salicylic acid in UGT74F2 wild-type (right) or T15S mutant (left). Omit electron density map (contoured at 1σ level) is shown in blue mesh. Average thermal parameters analysis suggests that occupancy of SA in the shown conformation (i.e. canonical SA from the SA/UDP complex of UGT74F2wt) is higher in wild-type than in the mutant.
